# Generalizable, Reproducible, and Neuroscientifically Interpretable Imaging Biomarkers for Alzheimer's Disease

**DOI:** 10.1002/advs.202000675

**Published:** 2020-06-09

**Authors:** Dan Jin, Bo Zhou, Ying Han, Jiaji Ren, Tong Han, Bing Liu, Jie Lu, Chengyuan Song, Pan Wang, Dawei Wang, Jian Xu, Zhengyi Yang, Hongxiang Yao, Chunshui Yu, Kun Zhao, Max Wintermark, Nianming Zuo, Xinqing Zhang, Yuying Zhou, Xi Zhang, Tianzi Jiang, Qing Wang, Yong Liu

**Affiliations:** ^1^ Brainnetome Center & National Laboratory of Pattern Recognition Institute of Automation Chinese Academy of Sciences Beijing 100190 China; ^2^ School of Artificial Intelligence University of Chinese Academy of Sciences Beijing 100049 China; ^3^ Department of Neurology the Second Medical Centre National Clinical Research Centre for Geriatric Diseases Chinese PLA General Hospital Beijing 100853 China; ^4^ Department of Neurology Xuanwu Hospital of Capital Medical University Beijing 100053 China; ^5^ Department of Radiology Tianjin Huanhu Hospital Tianjin 300350 China; ^6^ Brainnetome Center & National Laboratory of Pattern Recognition Institute of Automation Chinese Academy of Sciences Beijing 100190 China; ^7^ School of Artificial Intelligence University of Chinese Academy of Sciences Beijing 100049 China; ^8^ Center for Excellence in Brain Science and Intelligence Technology Institute of Automation Chinese Academy of Sciences Beijing 100190 China; ^9^ Department of Radiology Xuanwu Hospital of Capital Medical University Beijing 100053 China; ^10^ Department of Neurology Qilu Hospital of Shandong University Jinan 250012 China; ^11^ Department of Neurology Tianjin Huanhu Hospital Tianjin University Tianjin 300350 China; ^12^ Department of Radiology Qilu Hospital of Shandong University Jinan 250012 China; ^13^ State Key Laboratory of Management and Control for Complex Systems Institute of Automation Chinese Academy of Sciences Beijing 100190 China; ^14^ Department of Radiology the Second Medical Centre National Clinical Research Centre for Geriatric Diseases Chinese PLA General Hospital Beijing 100853 China; ^15^ Department of Radiology Tianjin Medical University General Hospital Tianjin 300052 China; ^16^ Beihang University Beijing 100191 China; ^17^ Department of Radiology Stanford University Stanford CA 94305 USA; ^18^ Pazhou Lab Guangzhou 510330 China

**Keywords:** Alzheimer's disease, computer‐aided diagnosis, neurobiological basis, neuroscientifically interpretable biomarkers, structural magnetic resonance imaging

## Abstract

Precision medicine for Alzheimer's disease (AD) necessitates the development of personalized, reproducible, and neuroscientifically interpretable biomarkers, yet despite remarkable advances, few such biomarkers are available. Also, a comprehensive evaluation of the neurobiological basis and generalizability of the end‐to‐end machine learning system should be given the highest priority. For this reason, a deep learning model (3D attention network, 3DAN) that can simultaneously capture candidate imaging biomarkers with an attention mechanism module and advance the diagnosis of AD based on structural magnetic resonance imaging is proposed. The generalizability and reproducibility are evaluated using cross‐validation on in‐house, multicenter (*n* = 716), and public (*n* = 1116) databases with an accuracy up to 92%. Significant associations between the classification output and clinical characteristics of AD and mild cognitive impairment (MCI, a middle stage of dementia) groups provide solid neurobiological support for the 3DAN model. The effectiveness of the 3DAN model is further validated by its good performance in predicting the MCI subjects who progress to AD with an accuracy of 72%. Collectively, the findings highlight the potential for structural brain imaging to provide a generalizable, and neuroscientifically interpretable imaging biomarker that can support clinicians in the early diagnosis of AD.

## Graphical abstract

This study proposes a deep learning model (3D attention network) to simultaneously capture imaging biomarkers with an attention mechanism module and advance the diagnosis of Alzheimer's disease. The generalizability and reproducibility are cross‐validated on independent databases with an accuracy up to 92%. Significant associations between the classification output and clinical characteristics of patients provide solid neurobiological support for the model.

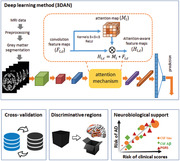

## 1 Introduction

Alzheimer's disease (AD) is the most prevalent cause of dementia, leading to irreversible brain damage. The disease is accompanied by memory deficits, communication difficulties, disorientation, and behavior changes and is a leading cause of death.^[^ ^1^ ^]^ Mild cognitive impairment (MCI), especially amnestic MCI, has a relatively high risk of conversion to AD and may be an intermediate state between healthy aging and dementia.^[^ ^2^, ^3^ ^]^ It is essential to identify underlying biomarkers or neuroimaging measures that can accurately capture clinical early diagnosis and quantify the stage of disease.^[^ ^4^, ^5^, ^6^ ^]^ However, despite decades of research, generalizable and reproducible biomarkers have not yet emerged.

Structural magnetic resonance imaging (sMRI) analysis provides an effective way to characterize anatomic abnormalities and the progression of AD, making it possible for medical scientists to identify imaging biomarkers of early neurodegeneration.^[^ ^7^, ^8^ ^]^ Existing structural MRI‐based studies have performed extensive morphometric analyses at the voxel level or region of interest (ROI) level, with the goal of quantifying the morphological characteristics of relevant regions in terms of volume, shape, and cortical thickness.^[6]^ However, statistical mapping methods can only characterize the detailed feature presentation of disease‐related changes from one perspective, such as volume or shape. Such neuroimaging biomarkers are modeled by compressing multi‐voxel imaging data into one or several values based on a pre‐determined ROI, a process which may have limited usefulness for individual diagnosis. To address this limitation, extracting high‐dimensional morphometric features has attracted increasing attention.^[^ ^9^, ^10^, ^11^, ^12^, ^13^ ^]^

Deep learning methods, especially convolutional neural networks, have been gradually applied to various medical image analysis tasks.^[^ ^14^, ^15^, ^16^, ^17^ ^]^ A convolutional neural network (CNN) can automatically learn the features that optimally represent the data. The CNN model, as a type of end‐to‐end architecture, can optimize both the representation of features and the classification performance based on a brain image. Conversely, the black box aspect of neural networks hinders us from obtaining valuable information about the focus of a network (i.e., the most discriminative localizations of brain abnormalities), which plays a key role in the diagnosis of disease. To this end, the attention mechanism module was developed to reveal the network on which to focus and to refine the feature representation and increase the representation power.^[^ ^18^, ^19^, ^20^, ^21^, ^22^ ^]^ Attention‐based networks have achieved successful applications in fields such as natural language processing,^[^ ^23^ ^]^ object detection,^[^ ^22^ ^]^ image classification, and synthesis.^[^ ^18^ ^]^

**Figure 1:**
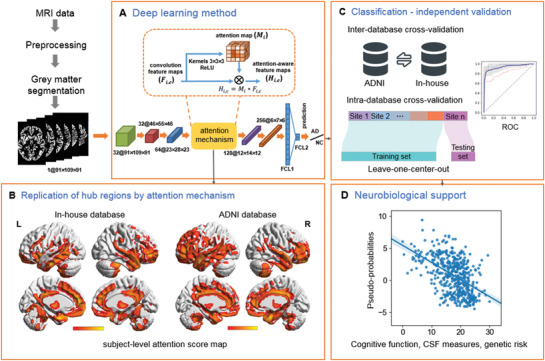
Schematic of the data analysis pipeline. A) The architecture of the 3D attention network (3DAN). In the attention mechanism module, each voxel *i* of the H × W × D‐dimensional feature maps *F*_*i*,*c*_ was weighted by the H × W × D‐dimensional attention map *M_i_*. The trainable attention map *M_i_* was independent of the channel of the features and was only related to the spatial position. B) The attention score map (left: in‐house database, right: ADNI database) was generated by the attention mechanism module of the 3DAN model, indicating the discriminative power of various brain regions for AD diagnosis. C) To test the robustness and generalizability of the 3DAN model, cross validations were performed using two completely independent databases (an in‐house database and the ADNI database) (Details can be found in Table 1). D) Investigation of the association between the classification output and clinical measures [that is the cognitive function measured by Mini‐Mental State Examination (MMSE), CSF beta‐amyloid (A*β*), CSF tau, and polygenic risk scores (PGRS)] in the AD and MCI groups.

Inspired by recent advances in attention‐based networks,^[^ ^20^, ^21^, ^22^ ^]^ we propose a 3D attention network (3DAN) that integrates an attention mechanism with a residual neural network (ResNet) to automatically capture the most discriminative localizations in brain images and jointly optimize the feature extraction and classifier performance for AD based on structural MRI images (**Figure** **1**). Because the extent to which brain structures are affected by AD varies,^[^ ^11^, ^24^, ^25^, ^26^ ^]^ we adopted an attention module to emphasize important atrophy localizations and suppress unnecessary ones along the spatial dimension. Based on the attention module, the discriminative localizations and refined feature representation were simultaneously learned in a data‐driven fashion. To test the robustness and generalizability of the imaging biomarkers for AD, cross validations were performed with two totally independent databases (*n* = 1832, in total). We also expected that the classification output would have a solid neurobiological basis. To investigate this hypothesis, we researched the association between the attention network output and clinical measures [that is the cognitive function measured by the Mini‐Mental State Examination (MMSE), CSF beta‐amyloid (A*β*), CSF tau, and polygenic risk scores (PGRS)] in the AD and MCI groups. Finally, we investigated whether the 3DAN could capture features that could predict the progression of disease.

## 2 Results

### 2.1 Diagnostic Performance

**Figure 2:**
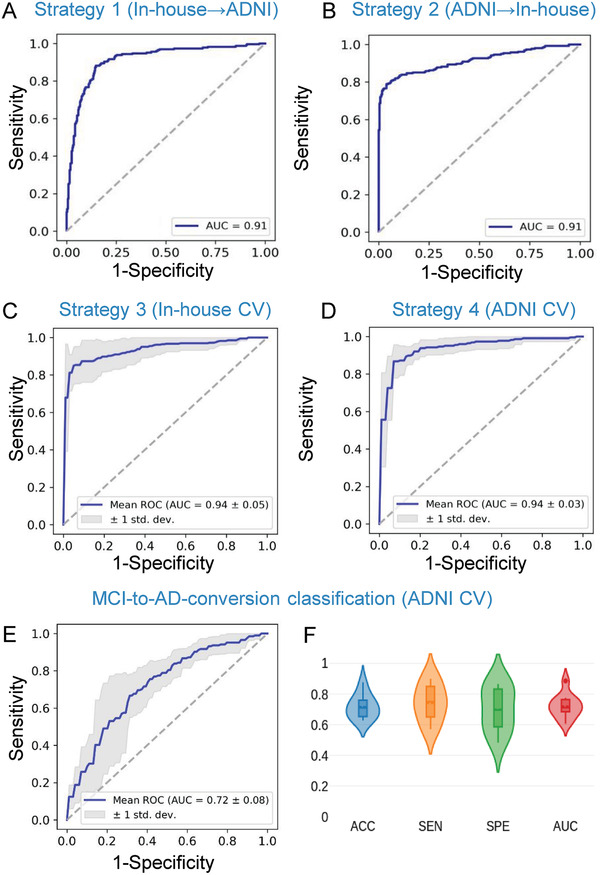
Diagnostic performance of the ROC curves for AD/NC classification (A–D) and pMCI/sMCI classification (E,F). A) ROC curve for the classifier that was trained on the in‐house database and tested on the ADNI database; B) ROC curve for the classifier that was trained on the ADNI database and tested on the in‐house database; C) ROC curve for the classifier that was trained and tested on the in‐house database with leave‐center‐out cross‐validation (CV); D) ROC curve for the classifier that was trained and tested on the ADNI database with tenfold cross‐validation; E) ROC curve of the pMCI/sMCI classification with tenfold cross‐validation on the ADNI database; F) violin plots for the distributions of the pMCI/sMCI classifications.

In total, 1832 subjects from our in‐house multi‐center database (*n* = 716) and the Alzheimer's Disease Neuroimaging Initiative (ADNI) database (*n* = 1116) were employed in this study (Table S1, Supporting Information). For the AD versus normal control (NC) classification, we conducted cross‐validations between the ADNI and the in‐house databases. For each strategy, one of the two databases was used as the training set and the other as the testing set. The classification accuracies were 86.1% (sensitivity (SEN) = 88.1%, specificity (SPE) = 84.6%, area under the curve (AUC) = 0.912) and 87% (SEN = 78.9%, SPE = 96.1%, AUC = 0.913) when taking the ADNI database and the in‐house database as the testing set, respectively (**Figure** **2**A,B, **Table** **1**). Then, we performed cross‐validations between the different scanners in the in‐house database using leave‐center‐out cross validation. The mean classification accuracy was 90.9% (SEN = 86.9%, SPE = 95.7%, AUC = 0.940, Figure 2C, Table 1). In the ADNI database, we performed a tenfold cross‐validation, and the mean classification accuracy was 92.1% (SEN = 89%, SPE = 94.4%, AUC = 0.941) (Figure 2D, Table 1; Figure S1, Supporting Information). For the progressive MCI (pMCI) versus the stable MCI (sMCI) classification, the mean classification accuracy was 71.7% (SEN = 74.0%, SPE = 70.1%, AUC = 0.721) using a tenfold cross‐validation on the ADNI database (Figure 2E,F).

**Table 1:** Classification performance of the proposed 3DAN method in the AD and NC classification tasks

	Training set	Testing set	ACC	SEN	SPE	AUC
**Strategy 1**	In‐house(***n*** = 716)	ADNI(***n*** = 1116)	0.861	0.881	0.846	0.912
**Strategy 2**	ADNI	In‐house	0.870	0.789	0.961	0.913
**Strategy 3**	In‐house leave‐center‐outcross‐validation		0.909	0.869	0.957	0.940
**Strategy 4**	ADNI 10‐foldcross‐validation		0.921	0.890	0.944	0.941

Abbreviations: ACC = accuracy; SEN = sensitivity; SPE = specificity; AUC = area under the curve of the receiver operating characteristic.

### 2.2 Important Regions Captured by the 3D Attention Network

**Figure 3:**
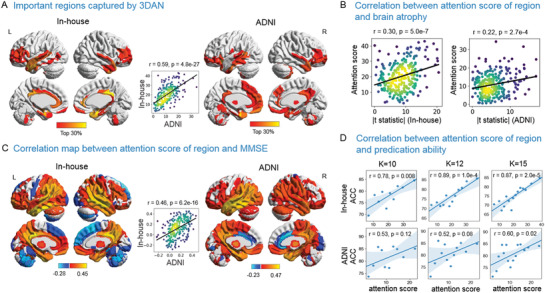
A) Mean attention score map derived from the in‐house database (left) and the ADNI database (right) and the correlation between these two attention maps (middle). Brighter colors indicate that the region is more discriminative for AD classification. The regions whose attention scores were in the top 30% (82/273) are displayed. The correlation figure indicates the replicability of the in‐house and ADNI databases. B) Correlation analysis between the mean attention score and the *t* statistic score of the gray matter volume between the NC and AD for the 273 ROIs in the Brainnetome Atlas for the in‐house database (left) and ADNI database (right). C) Correlation maps between the attention score for the regions and the MMSE scores with FDR correction (*p* < 0.05) in the in‐house database (left) and the ADNI database (right) and the relationship between the two correlation maps (middle). The correlation figure indicates the replicability of the in‐house and ADNI databases. D) Correlation between classification accuracy and the mean attention score of *K* groups of regions. The abscissa value of each point in the scatter plots represents the mean attention score of [273/*K*] brain regions in each group, and the ordinate value of each point in the scatter plots represents the classification accuracy based on the images of [273/*K*] brain regions in each group. At each *K*, the fact that higher attention scores are associated with higher classification accuracy reflects the effectiveness of the attention mechanism (Details of the method can be found in Figure S4, Supporting Information).

For each testing sample, by introducing the attention mechanism module, we obtained an attention value map, which indicated the discriminative power of various brain regions for AD diagnosis (**Figure** **3**A). The higher the value, the greater the discrimination ability of the region and the greater its potential as a biomarker. We resampled the mean attention map from an image size of 23 × 28 × 23, which is derived through two pooling operations (Figure 1A) on the original images for visualization. The attention network highlighted brain regions that were mainly located in the temporal lobe, hippocampus, parahippocampal gyrus, cingulate gyrus, thalamus, precuneus, insula, amygdala, fusiform gyrus, and medial frontal cortex (Figure 3A). More importantly, the attention pattern for the in‐house database and the ADNI database were significantly correlated (*r* = 0.59, *p* = 4.75e‐27, Figure 3A), which indicates the strong reproducibility of the results.

### 2.3 Attention Score for the Network is Associated with the Atrophy Pattern and MMSE Score

To evaluate whether the attention score changes were associated with brain alteration, we performed a correlation analysis between the significance of the group differences (T‐map) and the attention scores of regions derived from the network. The attention values had a significant correlation with the group difference map in the in‐house database (*r* = 0.30, *p* = 4.97e‐7) and the ADNI database (*r* = 0.22, *p* = 2.66e‐4), which indicates that the proposed model captured the features of the abnormal regions in AD (Figure 3B). We also repeated the above correlation analyses for the linear SVM model based on the Brainnetome (BN) atlas and found that the weights of the regions had no significant correlation with brain atrophy in either the in‐house database (*r* = 0.09, *p* = 0.14; Figure S2A, Supporting Information) or the ADNI database (*r* = 0.11, *p* = 0.07, Figure S2B, Supporting Information).

To evaluate whether the attention scores were associated with the patients’ cognitive abilities, regional correlation coefficients were calculated to measure the relationship between the attention scores and the Mini‐Mental State Exam (MMSE) scores in the AD and MCI groups. The attention scores of 220 (220/273 = 81%) and 210 (210/273 = 77%) brain regions were significantly associated with the MMSE scores (*p* < 0.05, FDR correction) for the in‐house and the ADNI databases, respectively. The high significance of this correlation indicates that the variability in the brain regions with higher attention scores in the subjects may reflect the degree of cognitive impairment of the subjects to some extent. These two MMSE‐associated maps of the in‐house and ADNI databases were significantly correlated (*r* = 0.46, *p* = 6.17e‐16, Figure 3C), which indicates that the results can be replicated across sites.

In addition, we preformed correlation analyses between the regional attention scores and the regional correlation coefficients to evaluate the overlap between the regions with higher attention scores and the regions whose attention score showed a significant correlation with the MMSE scores. The result showed that the greater the attention score for brain regions, the more significant the correlation between the attention score and the MMSE (*r* = 0.22, *p* = 2.73e‐4 for the in‐house database, *r* = 0.30, *p* = 3.77e‐7 for the ADNI database, Figure S3, Supporting Information). We also calculated the Dice similarity coefficient between the regions with a significant correlation with the MMSE score (regions in Figure 3C) and the top *n* regions with higher attention scores (*n* = 220 and 210 for the in‐house and ADNI databases, respectively). The results showed that regions with significant correlations between the attention scores and the MMSE had a wide overlap with the regions with higher attention scores (Dice coefficients = 0.83 and 0.82 for the in‐house and ADNI databases, respectively).

### 2.4 Effectiveness of the Attention Mechanism for Key Regions Identification

To evaluate the effectiveness of the attention module at capturing the key regions, we performed a correlation analysis between the attention score of a region and the classification accuracy of the model trained on those regions (Figure S4, Supporting Information). We carried out the experiments with a mean attention map obtained from models trained on the ADNI and in‐house databases, separately. There was a significant correlation between the attention score and classification performance (Figure 3D), which indicates that the key regions captured by the attention mechanism might be potential biomarkers for AD diagnosis.

### 2.5 Classification Probability is Related to Clinical Measures, Cognitive Functions, and Genetic Risk

**Figure 4:**
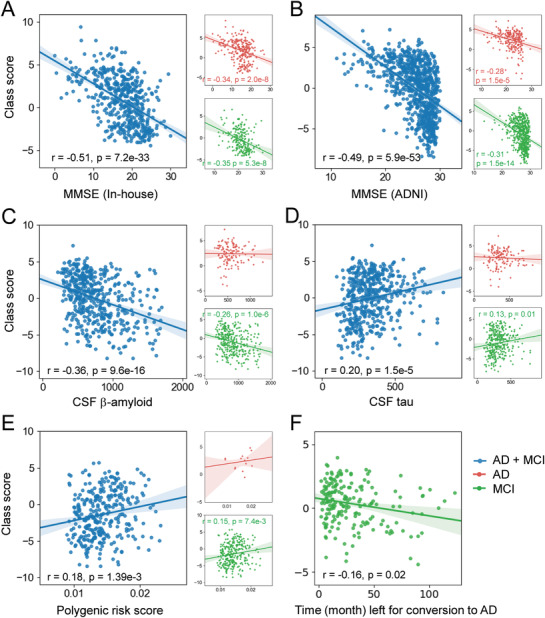
Correlations between the class scores and the MMSE scores in the in‐house database (A) and the ADNI database (B). Correlations between the class scores and the CSF A*β* (*n* = 472) (C), CSF tau (*n* = 472), (D) and polygenetic risk factors (*n* = 321) (E) of individual subjects in the ADNI database. F) Correlation between the classification output and the length of time before conversion to AD of the pMCI individuals in the ADNI database.

To investigate the clinical relevance of the prediction performance, we first performed a correlation analysis between the classification output and the MMSE scores of the AD and MCI groups (two groups together and separately) in the ADNI and in‐house databases. There was a significant correlation between the probability of AD predicted by the model and the cognitive impairment in the AD and MCI groups, with age and gender controlled (all *p* < 0.001, **Figure** **4**A,B). Then, we analyzed the correlation between the classification output and the CSF A*β* (*n* = 472), tau (*n* = 472), and PGRS (*n* = 321) using the ADNI database (details can be found in the Supporting Information). These results showed that the predicted probability of AD correlated significantly with the neuropathological changes and genetic factors (Figure 4C–E). There was a significant difference between the apolipoprotein E *ε*4 (APOE *ε*4) negative and APOE *ε*4 positive subjects in the MCI group, with age and gender controlled (*t*_341_ = 2.56, *p* = 0.01; Figure S5, Supporting Information). More importantly, we found that the more similar an MCI individual was to the AD group, the shorter the time to convert to AD (*r* = −0.16, *p* = 0.02, Figure 4F).

We also performed correlation analyses between the classification output of a linear SVM model based on the BN atlas and the MMSE score, CSF A*β*, tau, PGRS, and disease progression of the AD and MCI groups in the in‐house and ADNI databases. The classification output (i.e., individual pseudo‐probability of AD) was represented by the distances of the individual samples from the discrimination hyperplane. The results also showed significant correlations between the classification output and the neurobiological indices, MMSE scores, and disease progression (Figure S6, Supporting Information). Compared with the results of the linear SVM model, the individual pseudo‐probabilities of AD from our 3DAN model were more related to cognitive impairment, neurobiological changes, and genetic factors.

### 2.6 Comparison with Other Methods

From **Table** **2**, we can observe that the proposed method generally outperformed the VBM‐based and ROI‐based methods in the AD versus NC classifications for both the ADNI and the in‐house databases. It is worth noting that the in‐house database included subjects from different hospitals with different scanners. Although the heterogeneity between the data sources was great, the proposed method still achieved a classification accuracy of 87%. This indicates that the proposed method has good generalizability and robustness in dealing with independent datasets, factors which make this method very useful for clinical applications. The proposed method generally had better classification accuracies than VBM‐based (about 82%) and ROI‐based methods (about 81%) for AD diagnosis, which could be due to the richer feature representation power learned by the neural network.

**Table 2:** Comparison of the classification performance of the proposed 3DAN method with other methods for AD diagnosis in Strategies 1 and 2 in Table 1

Method	Training: In‐house, Testing: ADNI				Training: ADNI, Testing: In‐house			
	ACC	SEN	SPE	AUC	ACC	SEN	SPE	AUC
3DAN	0.861	0.881	0.846	0.912	0.870	0.789	0.961	0.913
ResNet	0.853	0.863	0.846	0.907	0.860	0.759	0.974	0.910
VBM	0.712	0.947	0.538	0.907	0.821	0.667	0.996	0.908
ROI‐AAL	0.720	0.947	0.551	0.885	0.811	0.651	0.991	0.888
ROI‐BNA	0.744	0.960	0.584	0.901	0.813	0.651	0.996	0.894

Abbreviations: ACC = accuracy; SEN = sensitivity; SPE = specificity; AUC = area under the curve of the receiver operating characteristic; BNA = Brainnetome Atlas; AAL = anatomical automatic labeling; ROI = region of interest; VBM = voxel‐based morphometric.

## 3 Discussion

To the best of our knowledge, this is the first attention‐based network to integrate automatic discriminative regions detection and classification into a unified framework for the identification of potential imaging biomarkers and the diagnosis of brain disease. The simple yet effective 3D attention network achieved a remarkable classification performance without handcrafted feature generation and model stacking when trained and tested on large‐ and multi‐scale analyses of databases (*n* = 1832) across sites and countries, highlighting the robustness and reproducibility of our proposed 3DAN method. It is also worth noting that the 3DAN introduced an attention mechanism to capture important atrophy localizations that are particularly associated with the diagnosis of disease. The classification output showed a strong association with the neurobiological indices (that is cerebrospinal fluid (CSF) amyloid *β* (A*β*), tau, and genetic risk scores). These results are highly advantageous for understanding the neurobiological architecture of a complex brain disease, thus making a step forward toward a precise early diagnosis of AD.

In terms of feature representation methods, the typical deep learning methods for AD, MCI, and normal control (NC) classification can be roughly categorized into four classes, including i) 2D slice‐level,^[^ ^15^, ^27^ ^]^ ii) 3D patch‐level,^[^ ^11^, ^28^ ^]^ iii) ROI‐level,^[^ ^29^ ^]^ and iv) 3D subject‐level.^[^ ^30^, ^31^, ^32^ ^]^ Contrary to conventional statistical mapping methods, few studies have provided valuable information about the discriminative localizations of images. Liu and colleagues proposed a two‐stage framework, by which the first stage could be used to identify discriminative landmarks at the patch‐based level and the second stage could be used to train a classifier using the selected patch‐based features.^[^ ^28^ ^]^ Lian and colleagues proposed a hierarchical, fully convolutional network to integrate the process of discriminative patches identification and the training of classification models into a unified framework for AD diagnosis.^[^ ^11^ ^]^ However, the potential for learning the discriminative localizations of the brain at the 3D subject level had not been well explored. Specifically, the proposed method introduced an attention module that highlights discriminative brain regions (such as the temporal lobe, hippocampus, parahippocampal gyrus, cingulate gyrus, and medial frontal cortex) at the subject level for AD diagnosis, and the post hoc analysis clearly showed that a higher attention score contributed significantly to the classification and that the attention maps had a high degree of replication (Figure 3A,D). The above related brain regions have been confirmed to be affected by AD pathology and are correlated with cognitive impairment in AD and/or MCI.^[^ ^33^, ^34^, ^35^ ^]^ As opposed to the conventional ROI‐ and voxel‐level based pattern analysis methods,^[^ ^36^, ^37^, ^38^, ^39^ ^]^ our proposed method does not require a priori information about pre‐defined brain regions. Basically, the method integrates a feature extraction process with a classification task and automatically learns discriminative features in a data‐driven fashion, leading to optimal classification performance.

Deep learning methods have increasingly been used in the computer‐aided diagnosis of AD due to their ability to learn to optimize feature representation and robustness. Thus, the most important advance offered by the present study is that the classifiers achieved high accuracy in the cross validation based on large independent multi‐site databases. Cross validation is very important for biomarker searching while the traditional leave‐one‐out or N‐fold cross validation using single site data is limited by relative smaller sample sizes, often leading to a poor generalization performance by the classifiers.^[^ ^40^, ^41^, ^42^, ^43^, ^44^, ^45^ ^]^ The proposed method achieved higher accuracy and a larger area under the curve (AUC) compared with the traditional support vector machine (SVM) classifiers based on ROI features in inter‐sites cross validations. The models trained here can be directly applied to new datasets; such generalizability is very important for future clinical translation.^[^ ^40^, ^41^, ^43^, ^46^, ^47^ ^]^ Another finding was that the classifier retained a relatively highly accurate prediction using only the baseline data to predict whether or not an MCI subject would convert to AD within three years, highlighting the effectiveness of the 3DAN and, therefore, its translational potential.

Critically, when we evaluated the relationship between the imaging measures and clinical features, the attention scores of the identified brain regions that were replicated across sites and between databases were significantly associated with the MMSE scores in these two databases. A highly significant positive correlation was also found between the attention scores and the pattern of atrophy in the gray matter (Figure 3B), which indicates that 3DAN captured the abnormal regions with a significant group difference between the AD and NC groups. Furthermore, there was a significant correlation between the classification output and the MMSE scores, revealing that the probability of an individual being classified as a patient with AD was correlated with the severity of the cognitive symptoms. AD patients are typically characterized by the presence of A*β*, tau neuritic plaques, and neurofibrillary tangles in the cerebral cortex.^[^ ^5^, ^48^ ^]^ The PGRSs are used to assess the cumulative genetic risk for a disorder,^[^ ^49^ ^]^ especially for AD,^[^ ^50^, ^51^, ^52^ ^]^ as confirmed by previous large‐scale genome‐wide association studies for the association between the PRGSs for AD and clinical markers (cognitive abilities, clinical evaluation, brain atrophy, tau, and A*β*).^[^ ^53^, ^54^, ^55^, ^56^ ^]^ More importantly, for a MCI individual, the closer the classification output was to that of the AD group, the more quickly the subject converted to AD. This finding provided additional evidence of the effectiveness of the 3DAN model. Hence, the significant associations between the classification output and the A*β*, tau, PGRS indices, and the length of time for MCI patients to convert to AD further provided a solid neurobiological basis for potential clinical applications of 3DAN.^[^ ^57^, ^58^ ^]^ And our further analyses showed that 5/6 of these correlations are stronger in 3DAN model than that in SVM (Figure S6, Supporting Information) highlighted the effectiveness of the attention mechanism for key regions identification and early classification.

While the experimental results emphasized promising potential clinical applications of the proposed method for AD studies, our study has some limitations that warrant consideration. First, the proposed method achieved good results in detecting important regions and diagnosing AD on two multi‐center databases. However, the performance and robustness of the proposed method should be further validated on a larger population before any actual clinical use.^[^ ^59^ ^]^ Second, we only employed one sMRI scan for each subject in the databases to explore the discriminative power of single mode data. It should be noted that in the present study the ADNI dataset was used to show the biological meaningfulness of the class scores, but the in‐house dataset only showed the disease severity associated alterations in the patient groups due to lack of other neurobiological measures. In the future, we need to introduce longitudinal and multimodal data, such as functional MRI, PET images, genetic data, and other techniques, to further improve the classification performance and understand the neurological basis(es) of AD.^[^ ^27^, ^60^, ^61^, ^62^ ^]^ The ability to predict which subjects had a higher risk of progression as well as the ability to detect earlier stages of AD is of great importance. Although we successfully classified pMCI and sMCI subjects with a classification accuracy of 72%, the classification performance for pMCI and sMCI needs further improvement using a large longitudinal dataset.^[^ ^63^ ^]^ Finally, higher resolution images and more advanced deep neural networks to improve the performance will be critical avenues for future studies.^[^ ^61^, ^64^, ^65^ ^]^

Overall, our proposed method is a novel model that integrates the attention mechanism into a deep CNN algorithm; we tested it in the largest neuroimaging analysis of AD to date. Without multi‐step feature selection and classification processes, the proposed end‐to‐end network achieved a better classification performance by leveraging the attention module. The interpretability of the 3DAN is conducive to promoting the clinical application of the deep learning algorithm for AD diagnosis. Our research team plans to investigate more specific neuroanatomical traits and AD phenotypes and explore sophisticated brain imaging measures to improve our understanding of AD and to increase early diagnosis and progression prediction.

## 4 Experimental Section

##### Study Design

Two sMRI databases were employed to cross validate the results in this study, 1) an in‐house multi‐center database; and 2) the Alzheimer's Disease Neuroimaging Initiative (ADNI) database (http://adni.loni.usc.edu). These databases contain baseline brain MR imaging from AD patients and normal controls (NC). This study was approved by the Medical Ethics Committee of the Institute of Automation, Chinese Academy of Sciences. The demographic information about the subjects in both the in‐house and ADNI databases are presented in Table S1, Supporting Information.

##### In‐house

The in‐house database consisted of 716 (261 AD, 224 MCI, and 231 NC) subjects imaged by six different scanners. These studies were approved by the medical ethics committees of the local hospitals. All the subjects or their legal guardians signed written consent forms and met identical stringent methodological criteria. Comprehensive clinical details can be found elsewhere in our previous studies.^[^ ^66^, ^67^, ^68^, ^69^, ^70^ ^]^ Detailed MRI acquisition protocol information for the different scanners is provided in the Supporting Information (Tables S1 and S2, Supporting Information).

##### ADNI

The data included in the present study consists of 1.5T or 3T T1‐weighted MR images acquired from a total of 1116 subjects (227 AD, 584 MCI, and 305 NC subjects). Of these subjects, 612 subjects had cerebrospinal fluid (CSF) amyloid‐*β* (A*β*), tau, and apolipoprotein E *ε*4 (APOE *ε*4) genotype measurements, and 536 subjects had polygenic risk scores (PGRS). The detailed information can be found in Tables S3 and S4, Supporting Information. In addition, MCI patients were further divided into progressive MCI (pMCI) subjects who converted to AD and stable MCI (sMCI) subjects who did not convert to AD. The subjects (203 and 295 sMCI) who had follow‐up data more than one year after the initial MRI and had consistent diagnoses for all their longitudinal scans were employed for pMCI/sMCI classification (Table S5, Supporting Information).

##### Image Pre‐Processing

To learn valuable information about regional changes in gray matter for the training model, structural MRI images were pre‐processed with the standard steps in the CAT12 toolbox (http://dbm.neuro.uni‐jena.de/cat/). All sMRI data were bias‐corrected, segmented into gray matter (GM), white matter (WM), and cerebrospinal fluid (CSF) and registered to Montreal Neurological Institute (MNI) space using a sequential linear (affine) transformation. The gray matter images were resliced to 2 mm × 2 mm × 2 mm cubic size, resulting in a volume size of 91 × 109 × 91 with 2 mm^3^ isotropic voxels.

##### Attention Based 3D Deep Learning Method

A simple yet effective 3D attention‐based network (3DAN) was proposed for AD diagnosis and to identify discriminative localizations.^[^ ^71^ ^]^ Specifically, the residual network (ResNet) ^[^ ^72^ ^]^ was used as the basic architecture. The 3DAN consisted of a convolutional layer, eight residual blocks, an attention mechanism module and a fully connected layer (Figure 1A). Each basic block consisted of two convolutional layers and each convolutional layer was followed by batch normalization, and a nonlinearity activation function ReLU.^[^ ^73^ ^]^ The sizes of the 3D feature maps were reduced from 91 × 109 × 91 to 46 × 55 × 46 to 23 × 28 × 23 by an average pooling function with a kernel size of 3 × 3 × 3. In the residual block, the output of each block *H* (*x*) adds the input *x* and the stacked nonlinear mapping of input *F*(*x*) directly through “shortcut connection” that addresses the degradation problem.

(1) H(x)=F(x)+x

The attention mechanism was carried out simply by a convolution layer with a set of filters of 3 × 3 × 3 kernel size.^[^ ^71^ ^]^ In the attention mechanism module, each voxel i of the H × W × D‐dimensional feature maps *F*_*i*,*c*_ was weighted by the H × W × D‐dimensional attention map *M_i_*. The trainable attention map *M_i_* was independent of the channel of the features and was only related to the spatial position (Figure 1).

(2) $$H_{i,c}=F_{i,c}*M_{i}$$

where, the spatial position (*x*, *y*, *z*) of the voxel is defined as i (i∈{1, …, H × W × D}, x ∈{1, …, H}, y∈{1, …, W}, z∈{1, …, D}) and c ∈ {1, …, C} is the index of the channel. The proposed network was implemented using Python based on the platform of Pytorch (version = 0.3.1). The code can be downloaded at https://github.com/YongLiuLab. The input is the normalized 3D gray matter density image and the output is a probability for each individual obtained by a soft‐max classifier trained with cross‐entropy loss. It is worth noting that this end‐to‐end network has no need of prior knowledge to design its selected features. The attention generation process for 3D feature maps has less computation overhead and adds little network complexity. The proposed network was optimized using the Adam algorithm with an initial learning rate of 10^−6^, and the batch size was set as 8.

##### Diagnostic Performance

To maximize the generalizability of the classifications, multiple classifiers that could provide individual‐level predictions of group status under four different cross‐validation strategies (Table 1) were created to evaluate the impact of pooling data across sites and training/testing at different centers. For the first strategy, the model was trained on an in‐house database and it was tested on the ADNI database. For the second strategy, subjects from ADNI were used as the training set, while subjects from in‐house database were used as an independent testing set. For the third strategy, the situation of inter‐site cross‐validation was considered, in which leave‐center‐out cross‐validation was conducted for the in‐house database between the different scanners. For the fourth strategy, the classifiers were trained and tested within the ADNI database using tenfold cross‐validation for discriminating the AD from NC (Table 1). In addition, the classifiers were trained and tested the classification performance for the pMCI versus sMCI classifications. For all the analyses, the accuracy, sensitivity, specificity, and area under the curve (AUC) of the receiver operating characteristic were used to evaluate the performance of our model.

##### The Link between the Network's Attention Score, Predication Ability, and Atrophy Pattern

To further evaluate the effectiveness of the attention module for capturing the key regions, the relationship between the attention score and the discriminative capacity of the brain regions involved in AD diagnosis was assessed. Specifically, the attention score for each of the 273 regions designated in the Brainnetome Atlas (http://atlas.brainnetome.org/) was first calculated. To reduce the number of repetitions needed for the retraining and classification process, the 273 brain regions were subdivided into *K* groups by sorting the attention scores. For groups 1 to *K* − 1, each group had [273/*K*] regions, and for group *K*, it had 273 − (*K* − 1)[273/*K*] regions (see Figure S4, Supporting Information). The mean attention score for each group of brain regions was calculated, so that each of the K groups had an attention score. To evaluate the classification capacity of each group, the gray matter density images of the brain regions represented by each group were used as the input for the network to retrain the classification model and recalculate the accuracy of the AD versus NC classification. The retraining and classification process was conducted *K* times. Finally, the Pearson's correlation coefficients between the classification accuracies and the attention scores for the K groups were calculated. To evaluate the generalization of the result, three different values were used for *K* (*K* = 10, 12, 15). This process was repeated three times (The details also can be found in Figure S4, Supporting Information).

In addition, a correlation analysis was performed between the significance of the group difference as indicated by a two‐sample *t* test and the attention scores of the regions derived from the network. Specifically, the mean gray matter density of 273 ROIs was calculated based on the Brainnetome Atlas and performed a two‐sample two‐side *t* test between the patients with AD and the NC group after regressing out the effects of age, gender, and site. Then, the Pearson's correlation coefficient between the absolute value of the *t* statistics and the mean attention score for the 273 ROIs in the in‐house database and in the ADNI database were calculated separately.

To validate whether the importance of the brain regions identified by the attention mechanism was associated with cognitive ability, the correlation between the attention scores and the MMSE scores for each ROI was also investigated, based on the Brainnetome Atlas, in the inter‐database cross validation strategies (the first and second strategies). The correlation between the two correlation maps that were obtained from the two databases to test the reproducibility was also calculated.

##### Correlation between Prediction Performance and Clinical Measures, Cognition, and Genetic Risk

To further assess the clinical relevance of the prediction performance, the correlations between the class score (i.e., individual pseudo‐probability of AD) and cognitive ability, neuropathological changes, or genetic risk factors of individual subjects in the ADNI and in‐house databases (here with the MCI subjects included to test whether a disease severity association exists) were investigated. The un‐normalized class scores from the 3DAN model were used, rather than the class posteriors returned by the soft‐max layer. The Pearson correlation coefficients were calculated between the classifier output and MMSE scores, CSF beta‐amyloid (A*β*), tau, or polygenic risk scores (PGRS) (details about the PGRS can be found in the Supporting Information) in individual subjects in the AD and MCI groups (also each group separately) after regressing out the effects of age and gender. We also evaluated the effect of the high risk APOE gene for the MCI group in the ADNI database by a two‐sample two‐sided *t* test between the two subgroups (APOE *ε*4+ versus APOE *ε*4‐) (*p* < 0.05). In addition, to test whether a disease conversion rate association could be identified, the relationship between the classifier output and the length of the time to convert to AD for the pMCI individuals in the ADNI database was also evaluated.

##### Methods for Comparison

The proposed 3D attention‐based method was compared with four conventional classification methods. These were 1) base ResNet architecture and 2) support vector machine (SVM) classifiers based on the anatomical automatic labeling (AAL) atlas, 3) SVM classifiers based on the Brainnetome (BN) atlas, and 4) SVM classifiers based on voxel‐level features. 1) The residual network architecture with 18 layers (ResNet‐18) was trained for AD diagnosis and included a convolutional layer, eight basic ResNet blocks, and a fully connected layer. Unlike our attention‐based method, the base ResNet did not integrate the attention mechanism into the network. SVM based on ROI features: Region‐level features were extracted from gray matter images based on 2) a coarse‐grained template (AAL atlas) and 3) a finer template (Brainnetome Atlas, BN atlas). The gray matter volumes of the respective 90 ROIs and 273 ROIs were separately quantified to train non‐linear support vector machine classifiers with a Gaussian RBF kernel. 4) SVM based on voxel‐level features: Considering the high dimension of the voxel‐based features, a statistical group comparison analysis was performed based on a *t* test to reduce the dimensionality and then constructed a non‐linear SVM classifier for disease classification.^[^ ^28^ ^]^

## Conflict of Interest

The authors declare no conflict of interest.

## Supporting information

Supporting Information
