# Supplementary material for: Generalizable, Reproducible, and Neuroscientifically Interpretable Imaging Biomarkers for Alzheimer's Disease
Source: Adv Sci (Weinh). 2020 Jun 9;7(14):2000675. doi: 10.1002/advs.202000675 (PMC7375255; doi:10.1002/advs.202000675)
Supplement: Supplementary file 1 — Supporting Information [file ADVS-7-2000675-s001.pdf]

Supplementary Materials for

Generalizable, reproducible, and neuroscientifically interpretable imaging biomarkers for Alzheimer's Disease

Materials and Methods 3

Description of in-house database 3

PL_G and PL_S 3

HH_Z 4

QL_W 4

XW_H 4

XW_Z 5

Polygenic risk scores in the ADNI database 6

Computation of the polygenic risk score 7

Supplement Results 8

Supplementary Figure 1. Violin plots for the distributions of the classification performance of AD vs. NC classification. (a) The results of cross-validation of six different scanners on the in-house database. (b) The results of 10-fold cross-validation on the ADNI database. 8

Supplementary Figure 2. Correlation between the weights of 273 regions derived from a linear SVM model based on the BN atlas and region atrophy in the in-house and ADNI databases. 9

Supplementary Figure 3. Correlation between the regional attention score and regional correlation coefficient between the attention score and the MMSE score in the in-house and ADNI databases. 10

Supplementary Figure 4. Schematic diagram for evaluating the correlation between attention value and discriminative ability of the brain regions for AD diagnosis. 11

Supplementary Figure 5. The result of a two-sample t test between subjects in the MCI group who had one or two APOE ε4 alleles and subjects without any APOE ε4 alleles. 12

Supplementary Figure 6. Correlations between the classification output of a linear SVM model based on the BN atlas and the MMSE score in the in-house and ADNI databases, and between the classification output and CSF Aβ, tau, PGRS, and disease progression in the ADNI database. 13

Supplementary Table 1. Demographic and clinical information of participants in the in-house and ADNI databases. 14

Supplementary Table 2. MRI scanner and image-acquisition protocol information for the in-house database. 15

Supplementary Table 3. Demographic and clinical information for the participants in the ADNI database whose data included the cerebrospinal fluid beta-amyloid, tau, and APOE ε4 genotype information. 16

Supplementary Table 4. Demographic and clinical information for participants who had polygenic risk score information in the ADNI database. 16

Supplementary Table 5. Demographic and clinical information about pMCI and sMCI in the ADNI database. 16

References 17

Materials and Methods

The MRI data used in this study are described in detail elsewhere as part of a study investigating altered spontaneous activity[1], altered brain networks [2] and changed hippocampus radiomics [3] in AD. Hence, in the supplemental material of several our previous papers, we have reported how we determined our sample, all data exclusions, all inclusion/exclusion criteria, whether inclusion/exclusion criteria were established prior to data analysis. Here, we provide only a very brief introduction of the data inclusion criteria, exclusion criteria, and acquisition methods as part of the supplemental material to maintain the scientific integrity of the present study.

Description of in-house database

PL_G and PL_S

This study was approved by the Medical Ethics Committee of PLA General Hospital. Written informed consent was obtained from each enrolled subject or his/her authorized guardian. All the participants were recruited by advertisement. Written consent forms were obtained from all the subjects or their legal guardians under protocols approved by the ethics committee of the Chinese PLA General Hospital. Prior to selection for this study, all the participants were given free physical, psychological, and laboratory examinations. After the assessments, all the patients received professional suggestions for further treatment.

All of the subjects were right-handed and underwent a battery of neuropsychological tests, including the MMSE, AVLT, Geriatric Depression Scale (GDS) [4], Clinical Dementia Rating (CDR) [5], and Activities of Daily Living (ADL) scale. In brief, the AVLT consisted of 1 learning trial in which a list of 10 Chinese double-character words was read, and the subject was asked to immediately recall as many items as possible. The trial was repeated twice, and the immediate recall score was the average of 3 accurate recalls. After a 5-minute delay, each subject was asked to recall the words from the initial list (AVLT-delayed recall). The subjects were then told to identify the 10 studied words that were inter-mixed with 10 novel words (AVLT-recognition).

The recruited AD patients fulfilled the following inclusion criteria: (1) diagnosed using the National Institute of Neurological and Communicative Disorders and Stroke and the Alzheimer’s Disease and Related Disorders Association criteria for probable AD; (2) CDR = 1 or 2; (3) currently receiving no nootropic drugs, such as cholinesterase inhibitors; and (4) able to perform the neuropsychological tests and tolerate MR scanning.

The diagnostic criteria for MCI were determined as previously described [6] and included the following: (1) memory complaints lasting at least 6 months; (2) CDR = 0.5; (3) intact functional status and ADL< 26; and (4) lack of dementia. The criteria for NC included the following: (1) normal physical status; (2) CDR = 0; and (3) without memory complaints.

Exclusion conditions for participants of this study included the following: (1) metabolic conditions such as hypothyroidism or vitamin B12/folic acid deficiencies; (2) psychiatric disorders such as schizophrenia or depression; (3) infarction or brain hemorrhaging, as indicated by MR/CT imaging; and (4) Parkinsonian syndrome, epilepsy, and other nervous system diseases that can influence cognitive function. In addition, patients with a metallic foreign body, such as a cochlear implant, heart stent, or other relevant MR scanning contraindications, were excluded from the study.

Related publications can be found elsewhere [7-17].

HH_Z

The dataset follows the protocol of PL_G and PL_S. This study was approved by the Medical Ethics Committee of Tianjin Huanhu Hospital. The patients were recruited from the memory clinic of the neurology department of Tianjin Huanhu Hospital, Tianjin, China. The control subjects were recruited from the local community using advertisements. Written informed consent was obtained from each enrolled subject or his/her authorized guardian. The participants underwent general physical, psychological, and laboratory examinations prior to enrollment in the formal study. The participants did not take medications that might have influenced cognition during the scans, and all patients received professional suggestions for further treatment.

QL_W

The dataset follows the protocol of PL_G and PL_S. This study was approved by the Medical Ethics Committee of Qilu Hospital of Shandong University. The patients were recruited from the memory clinic of the neurology department of the Department of Neurology and Radiology, Qilu Hospital of Shandong University, Jinan, China. The control subjects were recruited from the local community using advertisements. Written informed consent was obtained from each enrolled subject or his/her authorized guardian. The participants underwent general physical, psychological, and laboratory examinations prior to enrollment in the formal study. The participants did not take medications that might have influenced cognition during the scans, and all patients received professional suggestions for further treatment.

Related publication can be found elsewhere[18].

XW_H

The study was approved by the Medical Research Ethics Committee and Institutional Review Board of Xuanwu Hospital (ClinicalTrials.gov identifiers: NCT02353884 and NCT02225964). Part of the data have been used in several previous studies, and detailed information can be found elsewhere [19, 20].

All subjects underwent a series of standardized clinical evaluations, including a medical history interview, a neurologic examination, and a battery of neuropsychological tests. Neuropsychological tests included the Chinese version of the Mini-Mental State Examination (MMSE), the Beijing

version of the Montreal Cognitive Assessment (MoCA) [21][29], the Clinical Dementia Rating Scale (CDR)[5], the auditory verbal learning test (AVLT)[22], an activities of daily living (ADL) assessment, the Hachinski Ischemic Scale, the Hamilton Depression Rating Scale (HAMD) [23], and the Center for Epidemiologic Studies Depression Scale[24]. Confirmation of diagnosis for all subjects was made by the consensus of at least two experienced neurologists in the Neurology Department of Xuanwu Hospital. The diagnoses were based on the available data from the neuropsychological assessment evaluation, a battery of general neurological examinations, and subject symptoms as well as functional capacity reports.

Inclusion criteria for an MCI diagnosis included the following [25]: (a) memory complaints confirmed by an informant; (b) objectively impaired memory confirmed by neuropsychological tests; (c) a definite history of cognitive decline; (d) not meeting the criteria for dementia according to the Diagnostic and Statistical Manual of Mental Disorders, Fourth Edition, Revised (DSM-IV-R); and (e) a CDR score of 0.5.

AD subjects were diagnosed according to the National Institute of Aging-Alzheimer's Association (NIA-AA) criteria for clinically probable AD [26, 27]: (a) meeting the criteria for dementia; (b) insidious and gradual onset (not sudden) over more than 6 months; (c) definite history of declining cognition; (d) initial and most prominent cognitive deficits evident in amnestic or non-amnestic performance; and (e) hippocampal atrophy confirmed by structural MRI.

The NC patients were required to meet the following research criteria: (a) no memory concerns; (b) MMSE and MoCA scores within the normal range (adjusted for age, sex, and education); and (c) a CDR score of 0.

The exclusion criteria applied to all subjects included the following: (a) vascular cognitive impairment (Hachinski Ischemic Scale score > 4 points); (b) severe depression (HAMD score > 24 points or The Center for Epidemiological Studies Depression Scale score > 21 points); (c) other central nervous system diseases that could cause cognitive decline (e.g., epilepsy, brain tumors, Parkinson's disease, or encephalitis); (d) systemic diseases that could cause cognitive impairments (e.g., anthracemia, syphilis, thyroid dysfunctions, severe anemia, or HIV); (e) a history of psychosis or congenital mental growth retardation; (f) severe hypopsia or dysacusis; (g) cognitive decline caused by traumatic brain injury; (h) severe end-stage disease or severe diseases in acute stages; or (i) a history of stroke; or (j) unable to complete neuropsychological tests or with a contraindication for MRI.

XW_Z

All the participants were recruited by advertisement and supported throughout the testing procedures in a specialist neuropsychological research facility at Xuanwu Hospital, Beijing, China. Patients and informants (usually a family member) were interviewed clinically by a senior psychiatrist (X. Zhang). Written consent forms were obtained from all subjects or their legal guardians (usually a family member). The study was approved by the ethics committee of Xuanwu Hospital. AD subjects were diagnosed using standard operationalized criteria (DSM-IVR [American Psychiatric Association, 1994] and NINCDS-ADRDA [26]]).

Inclusion criteria for AD diagnosis included the following: Severity of dementia was assessed using the Clinical Dementia Rating (CDR) scale [5]. Patients with a diagnosis of AD and a CDR score of 1 were classified as mild AD; patients with a CDR score of 2 or 3 were diagnosed as severe AD.

Mild cognitive impairment (MCI) was diagnosed according to standard criteria [6, 28, 29], which included subjective memory loss with objective evidence of memory impairment in the context of normal or near-normal performance on other domains of cognitive functioning; minimal impairment of activities of daily living; and a CDR score of 0.5. Normal volunteers had a CDR score of 0.

All the participants satisfied the following inclusion criteria: (1) no history of an affective disorder within one month prior to assessment; (2) normal vision and audition; (3) able to cooperate with cognitive testing; (4) aged between 50 and 90 years; (5) no clinical history of stroke or other severe cerebrovascular disease; and (6) no more than one lacunar infarction, without patchy or diffuse leukoaraiosis, on neuroradiological assessment of conventional MR images.

The exclusion criteria included: (1) severe general medical disorders of the cardiovascular, endocrine, renal, or hepatic systems; neurological disorders associated with potential cognitive dysfunction, including local brain lesions, traumatic brain injury with loss of consciousness or confusion, and dementia associated with neurosyphilis, Parkinsonism, or Lewy body disease; psychiatric disorders including depression, alcohol or drug abuse; (2) concomitant use of psychotropic medication in large quantity; and (3) insufficient cognitive capacity to understand and cooperate with study procedures.

All the patients underwent a complete physical and neurological examination, an extensive battery of neuropsychological assessments, and standard laboratory tests. Healthy volunteers underwent a brief clinical interview and MMSE to confirm they satisfied the exclusion criteria for cognitive deficits, psychoactive drug use, and clinical disorders. The detailed information can be found elsewhere in our previous studies [30-34].

Polygenic risk scores in the ADNI database

PGRSs are used to assess the cumulative genetic risk for a disorder [35]. Previous results from large-scale genome-wide association studies found an association between the PRGSs for the AD patients and the structures of certain brain regions, amyloid, and CSF tau pathology [36-40].

We obtained whole-genome sequencing (WGS) data from 812 participants in the ADNI database and then performed a genotype quality control (QC) analysis using PLINK version 1.07 [41]. First, we removed individuals with missing genotype rates of greater than 0.05. In addition, we estimated the pairwise identity-by-descent (IBD) value to identify individuals with possible relative relationships. These identified pairs of individuals had more similar genotypes than expected by chance in a random sample. In each pair, the individual with more missing genotype information was removed. Next, we applied single nucleotide polymorphism (SNP)-level filtering, removing SNPs with a minor

allele frequency less than 0.01, SNPs missing rates greater than 0.05, and SNPs that significantly departed from Hardy-Weinberg equilibrium ($p < 0.001$). To control for population stratification, we carried out a principal component analysis (PCA) using GCTA version 1.91.4beta [42] on a linkage disequilibrium (LD) pruned set of autosomal SNPs obtained by carrying out LD pruning with PLINK and removing 5 long-range LD regions with the HapMap phase 3 reference data set [43]. We then obtained 10 principal components and excluded the outliers of the samples based on an SD >6 from the mean. Finally, we used SHAPEIT v2 (r790) [44] and IMPUTE2 [45] to impute ungenotyped SNPs from the 1000 Genomes Phase 1 reference dataset. Further analyses focused on autosomal SNPs with imputation quality scores greater than 0.8.

After the QC procedures, 792 ADNI subjects with more than 7 million SNPs were retained for subsequent analysis. After interleaving with the MRI image data, 536 subjects [NCs (N=215), MCI patients (N=307), AD patients (N=14)] with genome-wide single-nucleotide polymorphisms remained for analysis.

Computation of the polygenic risk score

We used the "score" utility in PLINK and recent summary statistics for AD [46] to compute the polygenic AD risk score. The polygenic risk score computing method was developed by Purcell and colleagues [35], as described by Holmes and colleagues [47]. Overall, eighteen PGRSs for each subject were obtained using different SNP inclusion thresholds, as follows: $P_T < 1$; $P_T < 0.5$; $P_T < 0.4$; $P_T < 0.3$; $P_T < 0.2$; $P_T < 0.1$; $P_T < 0.05$; $P_T < 0.01$; $P_T < 0.005$; $P_T < 0.001$; $P_T < 5\times10^{-4}$; $P_T < 1\times10^{-4}$; $P_T < 1\times10^{-5}$; $P_T < 1\times10^{-6}$; $P_T < 1\times10^{-7}$; $P_T < 1\times10^{-8}$; $P_T < 1\times10^{-9}$; and $P_T < 1\times10^{-10}$.

Choosing the PGRS under the appropriate SNP inclusion threshold is an important step. Specifically, we compared the polygenic risk scores for AD between the patients and controls and found that the difference was most significant when the threshold was equal to 1×10^{-6}, which suggested that this PGRS could best explain the patient-control difference in genetic structure. Therefore, the polygenic risk score with a threshold of 1×10^{-6} was used for further analysis.

Supplement Results

Supplementary Figure 1. Violin plots for the distributions of the classification performance of AD vs. NC classification. (a) The results of cross-validation of six different scanners on the in-house database. (b) The results of 10-fold cross-validation on the ADNI database.

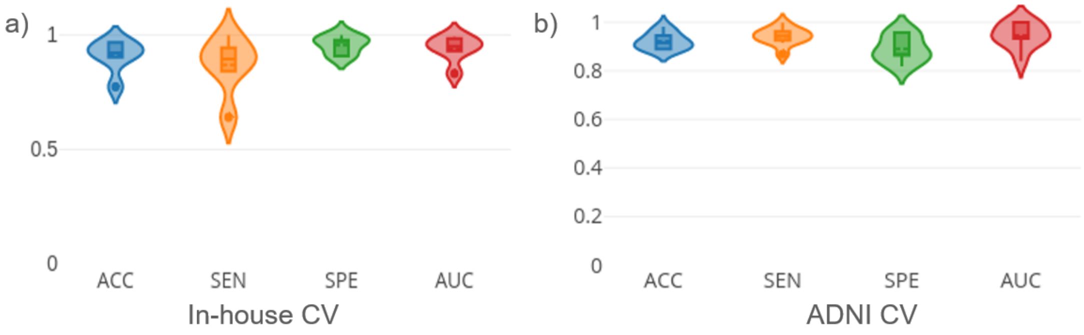

Supplementary Figure 2. Correlation between the weights of 273 regions derived from a linear SVM model based on the BN atlas and region atrophy in the in-house and ADNI databases.

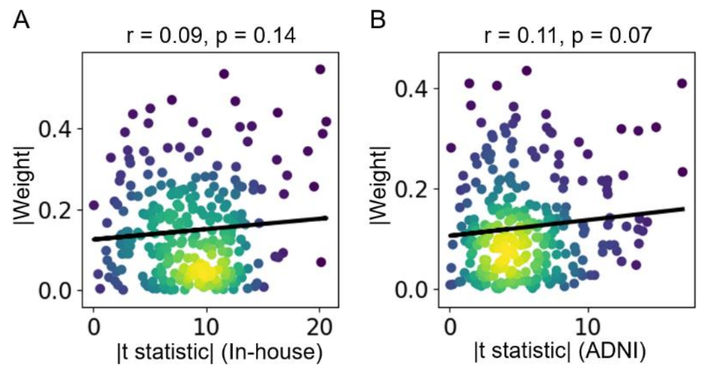

Supplementary Figure 3. Correlation between the regional attention score and regional correlation coefficient between the attention score and the MMSE score in the in-house and ADNI databases.

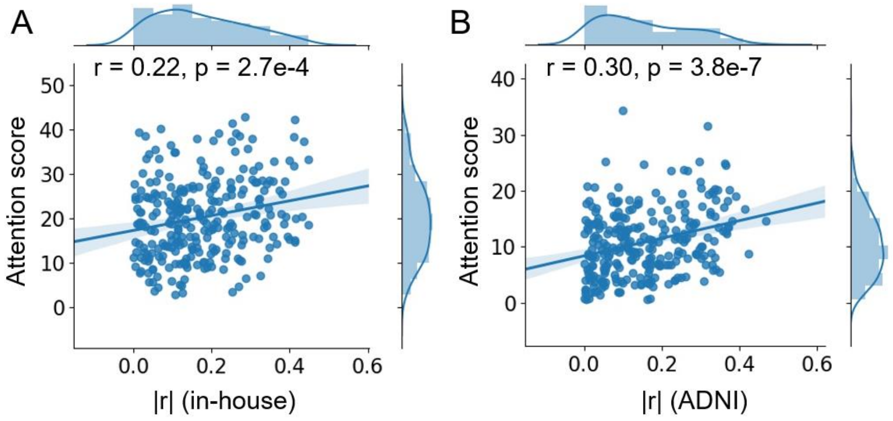

Supplementary Figure 4. Schematic diagram for evaluating the correlation between attention value and discriminative ability of the brain regions for AD diagnosis.

First, we divided the brain regions into K groups by sorting the attention values for the regions. For group 1, group 2, …, group K-1, each group had [273/K] regions, and for group K, it has 273-(K-1)*[273/K] regions. Then, we input the MRI images for each of these K groups into the network separately to retrain the classification model and recalculate the accuracy of the AD vs. NC classification. Finally, we calculated the Pearson's correlation coefficients between the classification accuracy values and the mean attention scores for the groups. A significant correlation indicates that the regions with higher attention scores made a greater contribution to the classification in the 3DAN model. Here, we set K = 10, 12, 15.

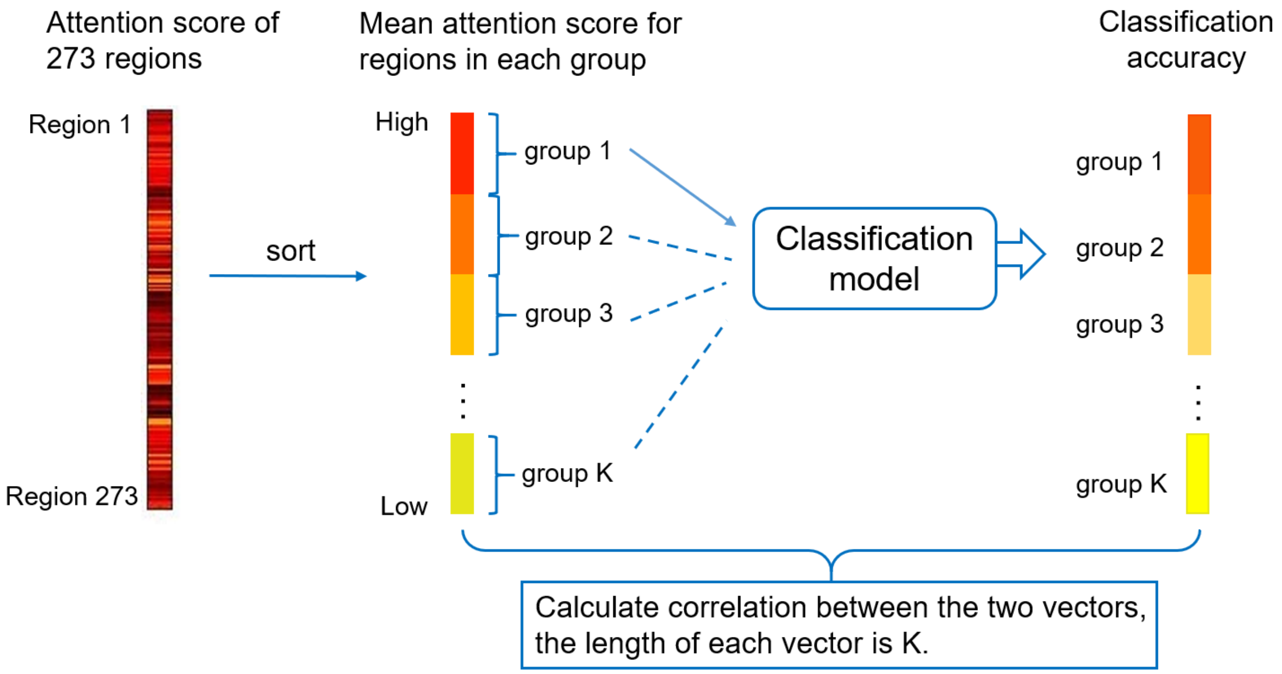

Supplementary Figure 5. The result of a two-sample t test between subjects in the MCI group who had one or two APOE ε4 alleles and subjects without any APOE ε 4 alleles.

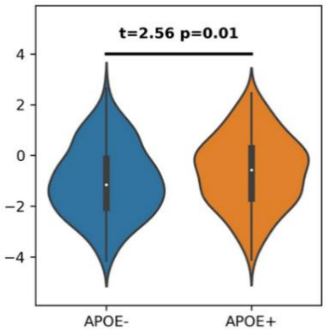

Supplementary Figure 6. Correlations between the classification output of a linear SVM model based on the BN atlas and the MMSE score in the in-house and ADNI databases, and between the classification output and CSF Aβ, tau, PGRS, and disease progression in the ADNI database.

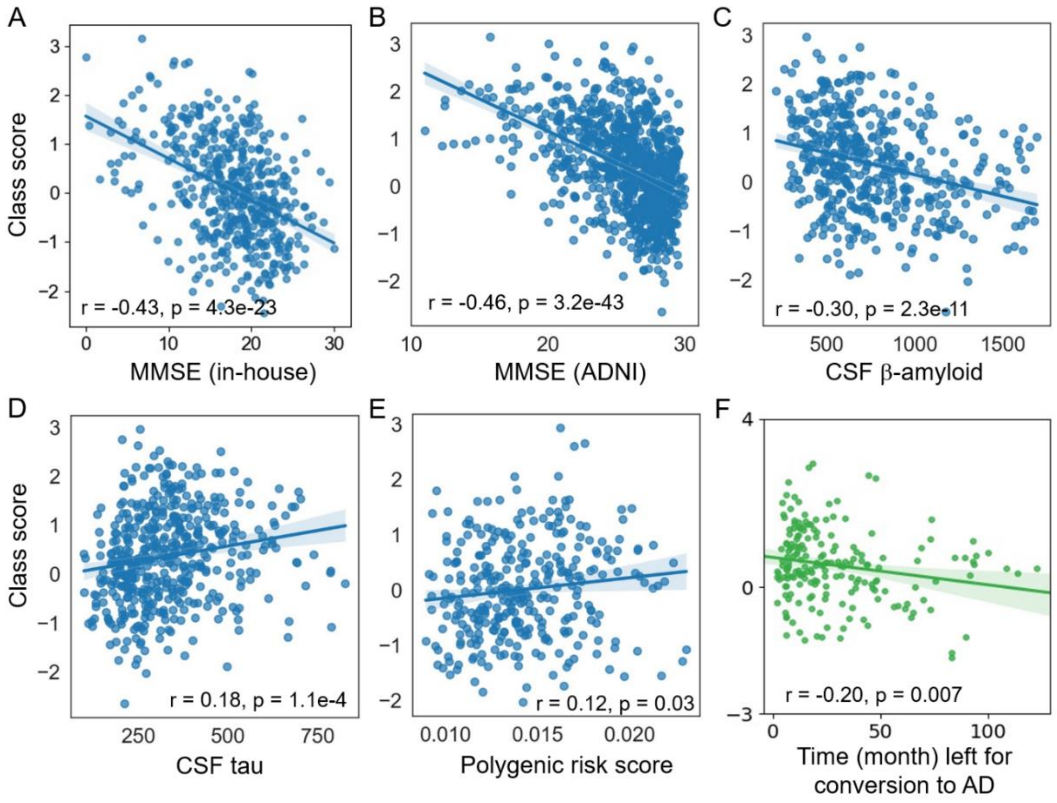

Supplementary Table 1. Demographic and clinical information of participants in the in-house and ADNI databases.

Database	Dataset	Group	Number	Age	Gender	MMSE
In-house (N=716)	HH_W	AD	36	67.3±8.2	18/18	15.8±5.6
		MCI	33	65.4±8.3	10/23	25.9±2.5
		NC	24	65.5±6.2	15/9	28.8±1.2
	PL_G	AD	38	72.0±9.4	25/13	19.1±4.6
		MCI	28	75.1±8.3	15/13	27.0±1.8
		NC	32	69.9±7.0	12/20	28.8±1.1
	PL_S	AD	38	71.7±8.3	22/16	17.6±5.6
		MCI	33	70.6±8.2	14/19	26.6±2.6
		NC	45	68.2±6.9	23/22	28.6±1.4
	QL_W	AD	64	67.9±7.2	37/27	19.8±2.9
		MCI	16	66.1±7.4	8/8	24.8±3.5
		NC	42	65.5±6.8	30/12	28.4±1.8
	XW_H	AD	47	69.1±8.5	31/16	16.7±6.4
		MCI	94	67.8±10.0	48/46	24.2±3.6
		NC	66	66.6±6.3	40/26	28.2±2.2
	XW_Z	AD	38	65.6±8.0	21/17	9.7±6.6
		MCI	20	70.7±8.5	11/9	21.3±5.5
		NC	22	65.5±8.2	14/8	28.3±1.5
ADNI (N=1116)		AD	227	74.8±7.6	105/122	22.0±3.5
		MCI	584	72.9±7.5	234/350	27.3±2.3
		NC	305	74.6±5.7	149/156	29.1±1.2

Supplementary Table 2. MRI scanner and image-acquisition protocol information for the in-house database.

Site	Field of strength	Brand	Number of head coil channels	Protocol name	Repetition time	Echo time	Flip angle	Field of view	Matrix	Slice number /thickness (no gap)
PL_S	3.0 T	Siemens Skyra	20	MP-RAGE	2530 ms	3.43 ms	90	256 × 256	256 × 256	192/1
PL_G	3.0 T	GE Signa HDx	8	FSPGR	7 ms	2.9 ms	90	240 × 240	256 × 256	166/1.2
HH_Z	3.0 T	Siemens Trio Tim	20	MP-RAGE	2000 ms	2.3 ms	90	232 × 256	232 × 256	192/1
QL_W	3.0 T	Siemens Verio	8	MP-RAGE	1900/2000 ms	2.3 ms	90	256 × 256	256 × 256	176/ 1
XW_H	3.0 T	Siemens Trio Tim	12	MP-RAGE	1900 ms	2.2 ms	90	256 × 224	512 × 448	176/ 1
XW_Z	3.0 T	Siemens Trio Tim	8	MP-RAGE	2000 ms	2.6 ms	90	256 × 224	256 × 224	176/ 1

One subject in HH_Z had FOV=222 mm×208 mm and matrix=256×240; one subject in HH_Z had FOV=216 mm×256 mm and matrix=216×256.

Supplementary Table 3. Demographic and clinical information for the participants in the ADNI database whose data included the cerebrospinal fluid beta-amyloid, tau, and APOE ε4 genotype information.

Group	Number	Age	Gender (F/M)	MMSE	CSF Aβ	CSF tau	ApoE ε4+	ApoE ε4-
NC	140	74.4±5.9	67/73	29.0±1.4	1013.3±374.8	227.2±90.4	95	45
MCI	342	72.5±7.4	137/205	27.4±2.3	857.4±339.7	284.2±134.7	178	164
AD	130	74.0±7.9	57/73	22.6±3.0	598.6±203.2	364.5±144.2	93	37

Supplementary Table 4. Demographic and clinical information for participants who had polygenic risk score information in the ADNI database.

Group	Number	Age	Gender (F/M)	MMSE	PGRS
NC	215	74.3±5.4	104/111	29.1±1.1	0.0137±0.0023
MCI	307	71.9±7.4	126/181	27.8±1.9	0.0143±0.0028
AD	14	74.6±6.7	9/5	23.3±1.9	0.0159±0.0004

Supplementary Table 5. Demographic and clinical information about pMCI and sMCI in the ADNI database.

Group	Number	Age	Gender (F/M)	MMSE
sMCI	295	72.8±7.6	122/173	27.6±2.3
pMCI	203	74.1±7.0	77/126	26.5±2.3

References

1. Li, J., et al., *ASAF: altered spontaneous activity fingerprinting in Alzheimer's disease based on multisite fMRI.* Science Bulletin, 2019. **64**(14): p. 998-1010.
2. Jin, D., et al., *Grab-AD: Generalizability and Reproducibility of Altered Brain activity and diagnostic classification in Alzheimer's Disease.* Hum Brain Mapp, 2020.
3. Zhao, K., et al., *Independent and reproducible hippocampal radiomic biomarkers for multisite Alzheimer's disease: diagnosis, longitudinal progress and biological basis.* Science Bulletin, 2020.
4. Yesavage, J.A., et al., *Development and validation of a geriatric depression screening scale: a preliminary report.* J Psychiatr Res, 1982. **17**(1): p. 37-49.
5. Morris, J.C., *The Clinical Dementia Rating (CDR): current version and scoring rules.* Neurology, 1993. **43**(11): p. 2412-4.
6. Petersen, R.C., et al., *Mild cognitive impairment: clinical characterization and outcome.* Arch Neurol, 1999. **56**(3): p. 303-8.
7. Feng, F., et al., *Radiomic Features of Hippocampal Subregions in Alzheimer's Disease and Amnestic Mild Cognitive Impairment.* Front Aging Neurosci, 2018. **10**: p. 290.
8. Alpert, K., et al., *The Northwestern University Neuroimaging Data Archive (NUNDA).* Neuroimage, 2016. **124**(Pt B): p. 1131-1136.
9. Zhou, B., et al., *Aberrant Functional Connectivity Architecture in Alzheimer's Disease and Mild Cognitive Impairment: A Whole-Brain, Data-Driven Analysis.* Biomed Res Int, 2015. **2015**: p. 495375.
10. Wang, P., et al., *Aberrant intra- and inter-network connectivity architectures in Alzheimer's disease and mild cognitive impairment.* Sci Rep, 2015. **5**: p. 14824.
11. Zhang, Z., et al., *Altered functional connectivity of the marginal division in Alzheimer's disease.* Curr Alzheimer Res, 2014. **11**(2): p. 145-55.
12. Yao, H., et al., *Longitudinal alteration of amygdalar functional connectivity in mild cognitive impairment subjects revealed by resting-state FMRI.* Brain Connect, 2014. **4**(5): p. 361-70.
13. Guo, Y., et al., *Grey-matter volume as a potential feature for the classification of Alzheimer's disease and mild cognitive impairment: an exploratory study.* Neurosci Bull, 2014. **30**(3): p. 477-89.
14. Zhou, B., et al., *Impaired functional connectivity of the thalamus in alzheimer' s disease and mild cognitive impairment: a resting-state FMRI study.* Curr Alzheimer Res, 2013. **10**(7): p. 754-66.
15. Yao, H., et al., *Decreased functional connectivity of the amygdala in Alzheimer's disease revealed by resting-state fMRI.* Eur J Radiol, 2013. **82**(9): p. 1531-8.
16. Wang, P., et al., *Perceptual and response interference in Alzheimer's disease and mild cognitive impairment.* Clin Neurophysiol, 2013. **124**(12): p. 2389-96.

17. Zhang, Z., et al., *Altered spontaneous activity in Alzheimer's disease and mild cognitive impairment revealed by Regional Homogeneity.* Neuroimage, 2012. **59**(2): p. 1429-40.

18. Wang, X., et al., *Amyloid-beta as a Blood Biomarker for Alzheimer's Disease: A Review of Recent Literature.* J Alzheimers Dis, 2019.

19. Yan, T., et al., *Rich club disturbances of the human connectome from subjective cognitive decline to Alzheimer's disease.* Theranostics, 2018. **8**(12): p. 3237-3255.

20. Li, S., et al., *Abnormal changes of multidimensional surface features using multivariate pattern classification in amnestic mild cognitive impairment patients.* J Neurosci, 2014. **34**(32): p. 10541-53.

21. Lu, J., et al., *Montreal cognitive assessment in detecting cognitive impairment in Chinese elderly individuals: a population-based study.* J Geriatr Psychiatry Neurol, 2011. **24**(4): p. 184-90.

22. Guo, Q., et al., *Norm of Auditory Verbal Learning Test in the Normal Aged in China Community.* Chinese Journal of Clinical Psychology, 2007. **15**(2): p. 132-134.

23. Hamilton, M., *A rating scale for depression.* J Neurol Neurosurg Psychiatry, 1960. **23**: p. 56-62.

24. Dozeman, E., et al., *The center for epidemiological studies depression scale (CES-D) is an adequate screening instrument for depressive and anxiety disorders in a very old population living in residential homes.* Int J Geriatr Psychiatry, 2011. **26**(3): p. 239-46.

25. Petersen, R.C., *Mild cognitive impairment as a diagnostic entity.* J Intern Med, 2004. **256**(3): p. 183-94.

26. McKhann, G., et al., *Clinical diagnosis of Alzheimer's disease: report of the NINCDS-ADRDA Work Group under the auspices of Department of Health and Human Services Task Force on Alzheimer's Disease.* Neurology, 1984. **34**(7): p. 939-44.

27. McKhann, G.M., et al., *The diagnosis of dementia due to Alzheimer's disease: recommendations from the National Institute on Aging-Alzheimer's Association workgroups on diagnostic guidelines for Alzheimer's disease.* Alzheimers Dement, 2011. **7**(3): p. 263-9.

28. Petersen, R.C., et al., *Current concepts in mild cognitive impairment.* Arch Neurol, 2001. **58**(12): p. 1985-92.

29. Choo, I.H., et al., *Topographic patterns of brain functional impairment progression according to clinical severity staging in 116 Alzheimer disease patients: FDG-PET study.* Alzheimer Dis Assoc Disord, 2007. **21**(2): p. 77-84.

30. Wu, Y., et al., *Distinct Changes in Functional Connectivity in Posteromedial Cortex Subregions during the Progress of Alzheimer's Disease.* Front Neuroanat, 2016. **10**: p. 41.

31. Liu, J., et al., *Impaired Parahippocampus Connectivity in Mild Cognitive Impairment and Alzheimer's Disease.* J Alzheimers Dis, 2016. **49**(4): p. 1051-64.

32. Liu, Y., et al., *Impaired long distance functional connectivity and weighted network architecture in Alzheimer's disease.* Cereb Cortex, 2014. **24**(6): p. 1422-35.

33. He, X., et al., *Abnormal salience network in normal aging and in amnestic mild cognitive impairment and Alzheimer's disease.* Hum Brain Mapp, 2014. **35**(7): p. 3446-64.

34. Song, J., et al., *Aberrant functional organization within and between resting-state networks in AD.* PLoS One, 2013. **8**(5): p. e63727.

35. International Schizophrenia Consortium, et al., *Common polygenic variation contributes to risk of schizophrenia and bipolar disorder.* Nature, 2009. **460**(7256): p. 748-52.

36. Gaiteri, C., et al., *Genetic variants in Alzheimer disease - molecular and brain network approaches.* Nat Rev Neurol, 2016. **12**(7): p. 413-27.

37. Logue, M.W., et al., *Use of an Alzheimer's disease polygenic risk score to identify mild cognitive impairment in adults in their 50s.* Mol Psychiatry, 2019. **24**(3): p. 421-430.

38. Tan, C.H., et al., *Polygenic hazard score, amyloid deposition and Alzheimer's neurodegeneration.* Brain, 2019. **142**(2): p. 460-470.

39. Axelrud, L.K., et al., *Polygenic Risk Score for Alzheimer's Disease: Implications for Memory Performance and Hippocampal Volumes in Early Life.* Am J Psychiatry, 2018. **175**(6): p. 555-563.

40. Tan, C.H., et al., *Polygenic hazard score: an enrichment marker for Alzheimer's associated amyloid and tau deposition.* Acta Neuropathol, 2018. **135**(1): p. 85-93.

41. Purcell, S., et al., *PLINK: a tool set for whole-genome association and population-based linkage analyses.* Am J Hum Genet, 2007. **81**(3): p. 559-75.

42. Yang, J., et al., *GCTA: a tool for genome-wide complex trait analysis.* Am J Hum Genet, 2011. **88**(1): p. 76-82.

43. Thorisson, G.A., et al., *The International HapMap Project Web site.* Genome Res, 2005. **15**(11): p. 1592-3.

44. Delaneau, O., J. Marchini, and J.F. Zagury, *A linear complexity phasing method for thousands of genomes.* Nat Methods, 2011. **9**(2): p. 179-81.

45. Howie, B.N., P. Donnelly, and J. Marchini, *A flexible and accurate genotype imputation method for the next generation of genome-wide association studies.* PLoS Genet, 2009. **5**(6): p. e1000529.

46. Jansen, I., et al., *Genetic meta-analysis identifies 10 novel loci and functional pathways for Alzheimer's disease risk.* bioRxiv, 2018: p. 258533.

47. Holmes, A.J., et al., *Individual differences in amygdala-medial prefrontal anatomy link negative affect, impaired social functioning, and polygenic depression risk.* J Neurosci, 2012. **32**(50): p. 18087-100.
